# Fucoidan, a Sulfated Polysaccharide, Inhibits Osteoclast Differentiation and Function by Modulating RANKL Signaling

**DOI:** 10.3390/ijms151018840

**Published:** 2014-10-20

**Authors:** Young Woo Kim, Seung-Hoon Baek, Sang-Han Lee, Tae-Ho Kim, Shin-Yoon Kim

**Affiliations:** 1 Department of Orthopedic Surgery, Kyungpook National University School of Medicine, Daegu 700-422, Korea; E-Mails: mujunggum2@lycos.co.kr (Y.W.K.); sbaek@knu.ac.kr (S.-H.B.); 2 Department of Food Science & Biotechnology, Kyungpook National University, Daegu 702-701, Korea; E-Mail: sang@knu.ac.kr; 3 Food & Bio-Industry Research Institute, Kyungpook National University, Daegu 702-701, Korea; 4 Skeletal Diseases Genome Research Center, Kyungpook National University, Daegu 700-721, Korea; 5 Biomedical Research Institute, Kyungpook National University Hospital, Daegu 700-721, Korea

**Keywords:** fucoidan, osteoclastogenesis, sulfated polysaccharide, bone resorption, RANKL (receptor activator of nuclear factor kappa B (NF-κB) ligand)

## Abstract

Multinucleated osteoclasts differentiate from hematopoietic progenitors of the monocyte/macrophage lineage. Because of its pivotal role in bone resorption, regulation of osteoclast differentiation is a potential therapeutic approach to the treatment of erosive bone disease. In this study, we have found that fucoidan, a sulfated polysaccharide extracted from brown seaweed, inhibited osteoclast differentiation. In particular, addition of fucoidan into the early stage osteoclast cultures significantly inhibited receptor activator of nuclear factor kappa B (NF-κB) ligand (RANKL)-induced osteoclast formation, thus suggesting that fucoidan affects osteoclast progenitors. Furthermore, fucoidan significantly inhibited the activation of RANKL-dependent mitogen-activated protein kinases (MAPKs) such as JNK, ERK, and p38, and also c-Fos and NFATc1, which are crucial transcription factors for osteoclastogenesis. In addition, the activation of NF-κB, which is an upstream transcription factor modulating NFATc1 expression, was alleviated in the fucoidan-treated cells. These results collectively suggest that fucoidan inhibits osteoclastogenesis from bone marrow macrophages by inhibiting RANKL-induced p38, JNK, ERK and NF-κB activation, and by downregulating the expression of genes that partake in both osteoclast differentiation and resorption.

## 1. Introduction

Adult bone mass is governed by tight regulation and balance between the osteoclast-mediated bone resorption and osteoblast-induced bone formation [1,2]. However, when osteoclastic bone resorption exceeds bone formation, the resulting imbalance causes bone-destructive diseases such as osteoporosis and rheumatoid arthritis [3]. Therefore, the activation of osteoclast is the target for novel therapeutic intervention for pathological bone loss.

Current drugs for bone health include inhibitors of osteoclastic-mediated bone resorption such as bisphosphonates, calcitonin, and estrogen that help to maintain bone mass and reduce fractures [4,5]. However, these pharmacological treatments have serious side effects such as hypercalcemia, increased risk of breast and endometrial cancer, and gastrointestinal intolerance against bisphosphonate [6,7]. Hence, safer alternatives that can be obtained from natural foods are being explored to treat osteoporosis.

Fucoidan is a sulfated polysaccharide that is found in brown seaweeds such as *Fucus vesiculosus*, *Ecklonia kurome*, and *Undaria pinnatifida*, and is often sold as commercial seafood in the East Asian countries. Fucoidan is mainly composed of l-fucose and sulfate, along with minor amounts of other sugars, including xylose, galactose, mannose, and glucuronic acid [8,9]. Fucoidan has a wide range of health benefits that include its role as an anti-coagulant, anti-thrombotic, anti-tumor, anti-viral, antioxidant, and anti-inflammatory factor [10,11,12,13,14]. Recent studies have shown that fucoidan promotes osteoblastic cell differentiation and bone biomaterial osteoconductive properties [15,16] while inhibiting adipocyte differentiation [17,18]. Other studies suggest that types A and B fucan sulfate, which were isolated from the sea cucumber, inhibited the formation of osteoclast-like cell formation [19].

In this study, we investigated the underlying mechanisms of how fucoidan effects osteoclast differentiation and activation.

## 2. Results

### 2.1. Inhibitory Effects of Fucoidan on Osteoclast Differentiation

**Figure 1:**
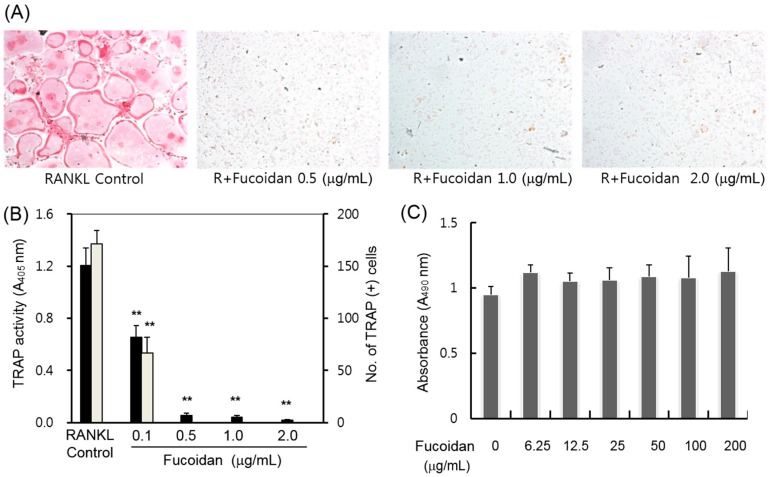
Effects of fucoidan on receptor activator of nuclear factor κB ligand (RANKL)-induced osteoclast differentiation in mouse bone marrow-derived macrophages (BMMs). (**A**) Mouse BMMs were cultured with macrophage colony-stimulating factor (M-CSF), RANKL, and various concentration of fucoidan. The osteoclasts were fixed and stained for TRAP; (**B**) Tartrate-resistant acid phosphatase (TRAP) activity was measured at 405 nm, and the cells positive for both TRAP and MNC (three or more nuclei) were scored as osteoclasts (** *p* < 0.05); (**C**) Cell viability was measured for BMMs cultured in the presence of M-CSF (30 ng/mL) and various concentrations of fucoidan for 2 days. Results represent the mean ± SD of three independent experiments.

We examined whether fucoidan inhibits RANKL-induced osteoclast formation in BMM cultures. The bone marrow-derived macrophages (BMMs) were induced to differentiate into osteoclasts in the presence of M-CSF and RANKL [3]. Mouse BMMs were cultured in the presence of RANKL and M-CSF together with or without various concentrations of fucoidan. Because both mononuclear osteoclast progenitor cells and osteoclast-like multinucleated cells (MNCs) are positive for TRAP, we examined whether fucoidan has any effect on the total TRAP activity and multinucleated cells (MNC) formation. As shown in Figure 1A,B, the treatment with 0.5 μg/mL fucoidan almost completely inhibited the formation of the multinucleated osteoclasts and TRAP activity. These inhibitory effects of fucoidan might be due to cytotoxicity or reduced growth of osteoclast progenitors. To exclude this possibility, we performed the cell proliferation assay. We observed that even very high concentrations of fucoidan (up to 200 μg/mL) did not cause any cytotoxic response in mouse BMM cells (Figure 1C).

### 2.2. Fucoidan Targets Early Stage Osteoclastogenesis

**Figure 2:**
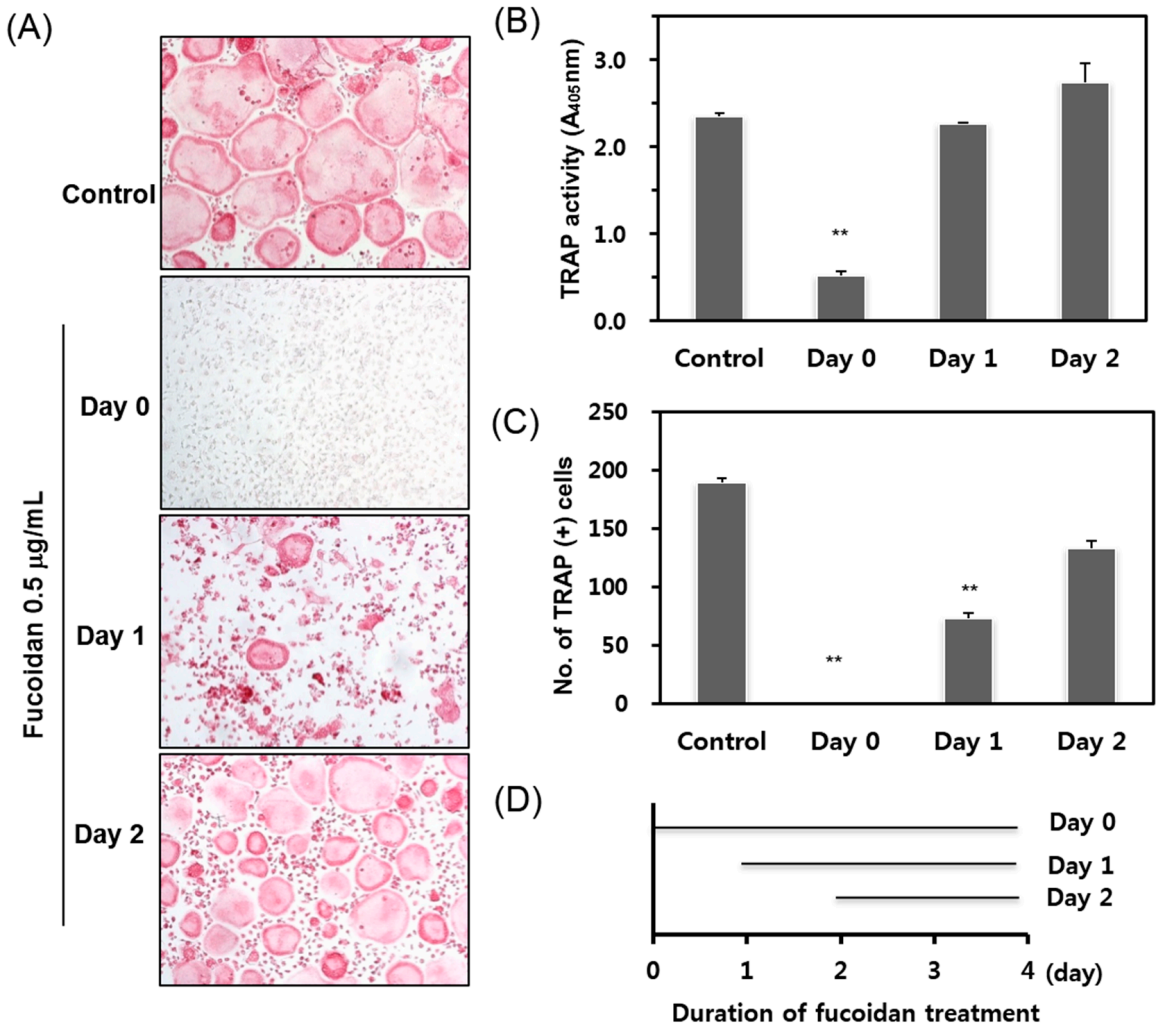
Effects of fucoidan on osteoclast differentiation treated for different time periods. (**A**) BMM cultures were treated with M-CSF and RANKL, and fucoidan (0.5 μg/mL) was added at indicated time points as shown in **D**. The cells were fixed and stained for TRAP; (**B**) TRAP activities and (**C**) TRAP + MNCs were scored. ** *p* < 0.05 represent significant differences from the relevant control.

Osteoclast differentiation is a multistep process that includes preosteoclast proliferation, multinucleation (cell fusion), and osteoclast activation (maturation). To determine the exact stage of osteoclastogenesis targeted by fucoidan, we added fucoidan into BMM cultures treated with RANKL and M-CSF at different time points (Figure 2A), and analyzed osteoclast formation on day 4. When fucoidan was added to the cells simultaneously with RANKL and M-CSF (Day zero; D0), osteoclast formation was completely abolished. By contrast, treatment with fucoidan on the second (Day one; D1) or on the third day (Day two; D2) after RANKL treatment failed to inhibit osteoclast formation. Most of the cells at D1 or D2 were TRAP-positive and multinucleated (Figure 2B,C). These results suggest that the fucoidan exerts its effect mainly during the early stage of osteoclastogenesis.

### 2.3. Fucoidan Down-Regulates RANKL-Induced Osteoclastogenesis-Related Genes

**Figure 3:**
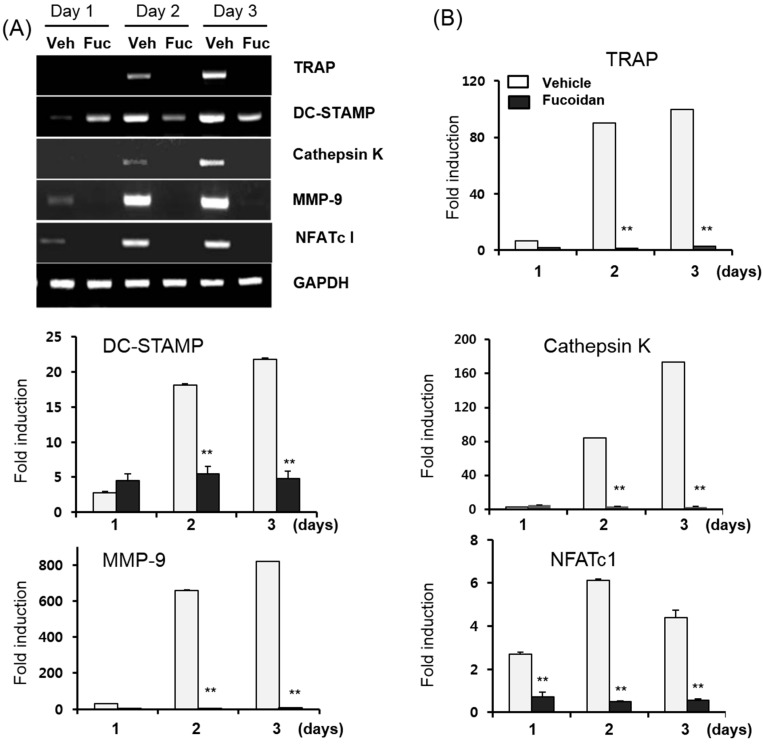
Effects of fucoidan on RANKL-induced gene expression during osteoclast formation. BMMs were cultured with 10 ng/mL M-CSF and 20 ng/mL RANKL in the presence or absence of 0.5 μg/mL fucoidan simultaneously. Total RNA was extracted at 1, 2 and 3 days and (**A**) RT-PCR and (**B**) quantitative real-time qPCR was used to analyze the mRNA expression levels of the indicated genes. GAPDH was used as a loading control. Data are expressed as the mean ± SD of triplicate samples. Veh: phosphate-buffered saline (PBS), Fuc: Fucoidan. ** *p* < 0.05 represent significant differences from the relevant control.

Osteoclast differentiation is associated with the up-regulation of specific genes in response to RANKL. To determine whether the inhibitory effect of fucoidan affects the expression of the osteoclast-specific genes, the mRNA expression of these was determined by semi-quantitative RT-PCR (Figure 3A) and real-time qPCR (Figure 3B). BMMs were cultured in the media containing M-CSF (10 ng/mL) and RANKL (20 ng/mL) in the presence or absence of 0.5 μg/mL fucoidan. In fact, fucoidan dramatically suppressed the expression of RANKL-induced osteoclast-associated genes such as *NFATc1*, *DC-STAMP*, *MMP-9*, *Cathepsin K*, and *TRAP* (Figure 3A,B). These data demonstrate that the fucoidan can reduce osteoclast differentiation by decreasing RANKL-induced osteoclast-specific genes.

### 2.4. Fucoidan Inhibits Bone Resorption in Vitro

**Figure 4:**
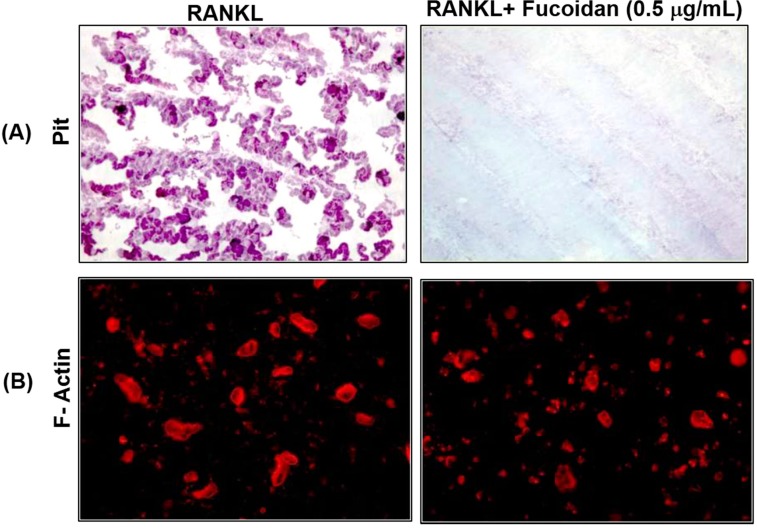
Effects of fucoidan on bone resorption and actin ring formation in mature osteoclasts. Mouse BMM cells were seeded on dentine slices and treated with RANKL (20 ng/mL) and M-CSF (10 ng/mL) to induce differentiation into osteoclasts. After osteoclasts had formed, the cells were treated with or without fucoidan (0.5 μg/mL) in the presence of M-CSF and RANKL for 48 h. (**A**) Resorption lacuna formation was examined by Hematoxylin staining; (**B**) Cells were fixed and stained with TRITC-phalloidin to visualize actin rings (lower).

Differentiated multinuclear osteoclasts undergo a morphological and functional polarization and begin to resorb mineralized bone surface. To examine whether fucoidan inhibits osteoclast function, we performed the *in vitro* resorption pit assay. Mature osteoclasts were incubated on a dentine slice with M-CSF and RANKL in the presence or absence of 0.5 μg/mL fucoidan for 2 days. As shown in Figure 4, staining for resorption pits using hematoxylin solution revealed that the fucoidan treatment significantly suppressed the formation of resorption lacuna compared with vehicle-treated cells. To resorb bone, osteoclasts must form a sealing zone with F-actin. When osteoclasts containing actin rings were treated with fucoidan for 2 days, significant differences in osteoclast were not observed as compared with that of the control. However, osteoclasts appeared smaller and irregularly shaped.

### 2.5. Fucoidan Down-Regulates RANKL-Induced C-Fos and NFATc1 Expression in BMMs

**Figure 5:**
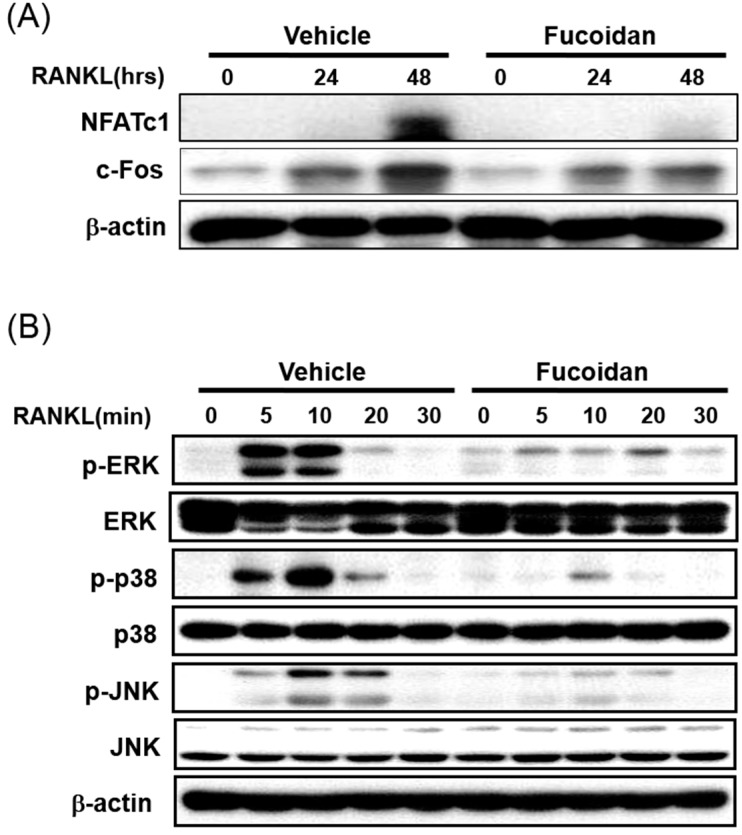
Effects of fucoidan on the activation of MAPKs and the expression of osteoclast-specific transcription factors by RANKL. BMMs were serum-starved for 5 h, pre-treated with fucoidan (5 μg/mL) or vehicle (PBS) for 4 h and then stimulated with RANKL (100 ng/mL). Cell lysates were collected, and equal amounts of protein were separated by 10% SDS-PAGE and analyzed by western blotting using (**A**) c-Fos and NFATc1 antibodies; and (**B**) anti-phospho-ERK, anti-phospho-p38, and anti-phospho JNK antibodies.

In order to understand the molecular mechanism underlying the inhibitory action of fucoidan during osteoclastogenesis in BMM culture, the effect of fucoidan on the expression of the key transcription factors, c-Fos and NFATc1, were examined. As reported previously, RANKL up-regulates the expression of c-Fos and NFATc1 in BMMs. Pretreatment of BMMs with fucoidan strongly inhibited both RANKL-induced mRNA expression of NFATc1 (Figure 3) and protein expression of c-Fos and NFATc1 when compared with that of untreated control (Figure 5A). These results demonstrated that the inhibitory effects of fucoidan involve inhibition of major transcription factors such as c-Fos and NFATc1.

### 2.6. Fucoidan Inhibits the RANKL Induced Phosphorylation of MAPKs in BMMs

To elucidate the molecular mechanisms underlying the inhibitory action of fucoidan on RANKL-induced c-Fos and NFATc1 expression, we examined whether fucoidan affects RANKL-induced early signaling molecules such as ERK, JNK, and p38. As shown in Figure 5B, RANKL-induced phosphorylation of these MAPK signaling molecules reached a maximum within 10 min, and then returned to the basal level. Pretreatment with BMMs with 5 µg/mL fucoidan for 4 h significantly decreased the phosphorylation of ERK, p38, and JNK compared with that of the controls (Figure 5B). Together, these results suggest that the fucoidan exerts its inhibitory function on the signaling molecules that lead to the activation of MAPK pathways.

### 2.7. Fucoidan Inhibited RANKL Induced Nuclear Transport of NF-κB

**Figure 6:**
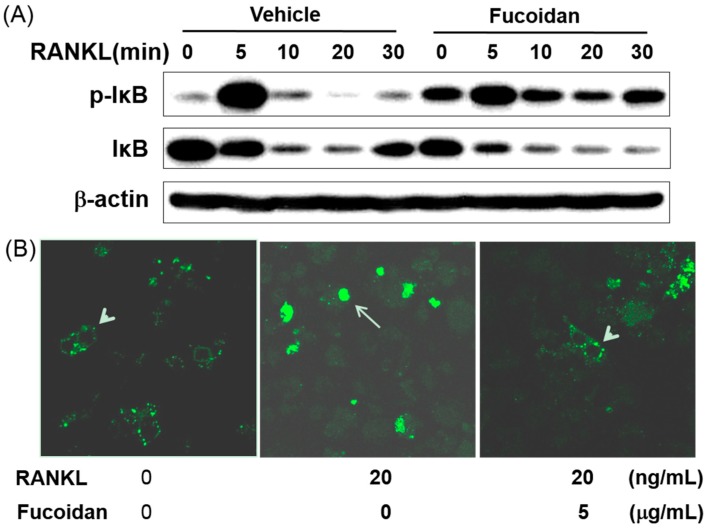
Fucoidan inhibits osteoclast differentiation via suppression of NF-κB activation. (**A**) BMMs were serum-starved for 5 h, pre-treated with fucoidan (5 μg/mL) or vehicle (PBS) for 4 h and stimulated with RANKL (100 ng/mL) for the indicated time points. Cell lysates were prepared and subjected to western blotting with phospho-IkB antibody. The membrane was stripped and re-probed with anti-IκB and anti-actin antibodies; (**B**) Starved cells were pre-treated with fucoidan (5 μg/mL) prior to RANKL (100 ng/mL) addition, and the localization of the p65 subunit of NF-κB was determined by immunofluorescence. From left to right, the image shows cytoplasmic localization of NF-κB (arrowheads), and nuclear localization of NF-κB (arrow) in unstimulated and RANKL stimulated cells, respectively.

In addition to MAPKs, the activation of transcription factor NF-κB is also important in osteoclast differentiation [2,20]. Phosphorylation of IκB, followed by its degradation is required for the activation of NF-κB. Therefore, we analyzed these parameters in BMMs with RANKL in the presence or absence of fucoidan. RANKL stimulated the phosphorylation of IκB with a maximum level at 5 min (Figure 6A, lane 2), followed by IκB degradation within 10–20 min (Figure 6A, lanes 3, 4 and 5). Treatment with fucoidan delayed the RANKL-induced IκB phosphorylation, as well as decreased the level of phosphorylation in BMMs (Figure 6A, lanes 5 and 10). To confirm whether degradation of IκB and/or nuclear translocation of NF-κB were mediated by fucoidan, we performed immunofluorescence staining of the p65 subunit of NF-κB using a pLL-1-NF-κB p65 GFP vector kit. The p65 subunit uniformly localized in the cytoplasm in unstimulated cells. Interestingly, RANKL treatment distinctly translocated the p65 to the nucleus in BMMs, and this translocation was inhibited by treating with 5 μg/mL fucoidan (Figure 6B). Thus, fucoidan inhibits NF-κB activation either through proteasomal degradation of IκB or by directly inhibiting RANKL-induced translocation of the p65 subunit.

## 3. Discussion

Osteoclasts are multinucleated cells that have a unique role in bone degradation. Abnormal increases in the RANKL signaling cascade enhances osteoclast development and bone resorption activity, which in turn is associated with various bone diseases such as osteoporosis, metastatic cancers, and osteolytic bone destruction. Therefore, the inhibition of osteoclast formation and/or its activity may be a useful approach for the treatment of pathological bone disorders. In this study, we report that fucoidan dramatically inhibited TRAP activity and multinucleated cell formation (Figure 1A,B) while eliciting no cytotoxic responses up to 200 μg/mL fucoidan in mouse BMMs (Figure 1C). Osteoclastogenesis consists of multiple steps including cell adhesion, differentiation, fusion, and activation. To determine which stage of osteoclastogenesis was targeted, fucoidan was added into the RANKL-treated BMMs at various time points. The TRAP activity and osteoclast formation inhibited by fucoidan treatment occurred only in the initial stage, and not in the middle or terminal stages of the culture (Figure 2). These results suggest that the fucoidan primarily acts on osteoclast precursors. Accordingly, the expression of osteoclast specific genes such as *NFATc1*, *DC-STAMP*, *MMP-9*, and *TRAP* was reduced in the presence of fucoidan (Figure 3).

Several transcription factors including PU.1, NF-kB, c-Fos, and NFATc1 are pivotal the development of osteoclast from its precursors [21,22]. In particular, the c-Fos/c-Jun/NFATc1 pathway is essential during osteoclast development and the lack of any of these three components arrests osteoclastogenesis [23]. The c-Fos-deficient mice exhibit a severe osteopetrosis due to the failure of osteoclast differentiation [22,24]. Furthermore, RANKL-induced NFATc1 expression is abrogated in the c-Fos-deficient mice [25,26] implying that NFATc1 acts downstream from c-Fos during osteoclast differentiation. In our study, fucoidan significantly inhibited the mRNA and the protein expression of NFATc1 (Figure 3 and Figure 5A), indicating that fucoidan targets NFATc1 to inhibit osteoclast formation.

NFATc1 and c-Fos are also involved in early signaling pathways, including mitogen-activated protein kinases (MAPKs) and NF-κB. Stimulation of RANKL has been reported to activate three well known MAPKs: ERK, JNK, and p38 [27]. In addition, SP600125, a JNK inhibitor, and SB203580, a p38-MAPK pathway specific inhibitor strongly inhibits osteoclastogenesis [21,28]. In this study, we found that the RANKL-induced phosphorylation of ERK, p38, and JNK decreased in response to the fucoidin treatment in murine BMMs (Figure 5B), suggesting that the inhibition of MAPK pathway is possible in the inhibitory action of this agent.

Activation of the NF-κB pathway is a prerequisite for osteoclast differentiation. Several genetic studies demonstrate a crucial role of NF-κB signaling in osteoclastogenesis, including p50/p52 NF-κB double-knockout mice that displayed severe osteopetrosis, due to impaired osteoclast differentiation [20,29]. In addition, NF-κB has been reported to function upstream of c-Fos and NFATc1 [30]. Prior to any stimulation, NF-κB is locked in an inactive state by the inhibitory IκB proteins. Exposure to RANKL triggers IκB phosphorylation and proteasome-mediated degradation of IκB allowing the nuclear translocation of NF-κB in BMMs [31]. In this study, fucoidan treatment decreased the degradation of IκB and attenuated the RANKL-dependent activation of NF-κB (Figure 6). Nuclear translocation of NF-κB complex (p65/p50/c-Rel), a hallmark for molecular osteoclastogenesis, was evident through the p65 immunofluorescence. The p65 of unstimulated BMMs showed uniform localization in the cytoplasm. In contrast, RANKL-treated BMMs showed distinct nuclear translocation, which was inhibited by the fucoidin treatment, indicating that the fucoidan inhibits osteoclastogenesis either by impairing proteasomal degradation of IκB or by directly inhibiting RANKL-induced p65 translocation. Thus, these results suggest that the suppression of RANKL-induced MAPK and/or delayed NF-κB activation in the presence of fucoidan contributes to its potent inhibition of c-Fos and NFATc1.The formation of an actin ring structure in bone resorbing osteoclasts is essential for bone resorption by activated osteoclasts [32,33]. Therefore, finding drugs that disrupt the integrity of the actin ring could be a useful therapeutic approach for reducing bone resorption. In this study, fucoidan inhibited bone-resorbing activity of mature osteoclasts (Figure 4). Despite abnormal osteoclast size and shape, the number of osteoclasts was comparable with that of the control group. Moreover, fucoidan did not disrupt the RANKL-induced actin ring in these osteoclasts. These results suggest that mature osteoclast apoptosis or disruption of actin ring formation may not be inherent to fucoidan’s inhibition of osteoclastic bone resorption.

Fucoidan mainly composed of l-fucose and sulfate, together with minor amounts of other sugars, including xylose, galactose, mannose, and glucuronic acid [8,9]. Despite intensive research, the exact correlation between the bioactivity and the structural molecular features of fucoidan, which vary depending on seaweed species and extraction methodology, has yet to be clarified. However, the important structural issues for bioactivity appear to include the degree of sulfation and the size of the molecules [34,35]. Fucoidan isolated from *U. pinnatifida* has a higher sulfate and l-fucose content and more bioactivities than that extracted from other brown seaweeds [9,36,37]. The sulfate content of fucoidan used in this study was minimum 25%, as sulfur. Previously, the effect of several sulfated glycosaminoglycans (GAGs), another type of biologically active sulfated polysaccharide, on osteoclastogenesis *in vitro* were reported. Ariyoshi *et al.* [38] and Shinmyouzu *et al.* [39] showed an inhibition of osteoclastogenesis after a direct interaction of GAGs with RANKL. GAGs are long-chain compounds composed of repeating disaccharide units with a carboxyl group and one or more sulfates, in which one sugar is *N*-acetylgalactosamine or *N*-acetylglucosamine. Heparin and chondroitin sulfate E inhibited osteoclast formation and function [39,40,41]. Theoleyre *et al.* [41] reported that heparin binds to osteoprotegerin (OPG), a decoy receptor for RANKL, with a high-affinity and that preincubation of OPG with heparin inhibited the binding of OPG to the RANKL–RANK complex. Dermatan sulfate (DS) also strongly bound to RANKL and completely inhibited RANK binding to RANKL, and subsequently suppressed osteoclast formation [39]. The RANK–RANKL binding causes the phosphorylation of p38 MAPK and ERK, and that such phosphorylation leads to osteoclast differentiation [42]. These findings suggest that the sulfated portion of fucoidan may important for inhibiting osteoclastogenesis. In this study, we found that the RANKL-induced phosphorylation p38 mainly decreased in response to the fucoidin treatment in murine BMMs. Therefore, binding of fucoidan either to RANKL, RANK or both that could impair an active receptor/ligand-complex formation would be another possible mechanism of inhibition.

As is well known, the bioactive properties of fucoidan may vary depending on the source of seaweed, the compositional and structural traits, the content of sulfate and purity. Hence further studies should be focused on standardization including administration method, necessary dosages to reach their target as an oral or intravenous drug or as part of biomaterials.

## 4. Materials and Methods

### 4.1. Chemicals and Reagents

Receptor activator of nuclear factor κB ligand (RANKL) and macrophage colony-stimulating factor (M-CSF) were purchased from R&D Systems (Minneapolis, MN, USA). Fucoidan derived from *Undaria pinnatifida*, a tartrate-resistant acid phosphatase (TRAP) staining kit, and β-actin were bought from Sigma-Aldrich (Acid phosphatase kit 387-A, St. Louis, MO, USA). Specific antibodies against phospho-ERK1/2 (Thr202/Tyr204), phospho-JNK1/2 (Thr183/Tyr185), phospho-p38 (Thr180/Tyr182), phospho-IκB (Ser32), IκB, and c-Fos were purchased from Cell Signaling Technology (Danvers, MA, USA). Antibodies against ERK, JNK, p38 (Thr180/Tyr182) were purchased from Santa Cruz Biotechnology, Inc. (Santa Cruz, CA, USA). NFAT*c*1 and dentine slices were purchased from BD Biosciences (Franklin Lakes, NJ, USA), and Immunodiagnostic Systems Limited (Tyne & Wear, UK) respectively. All other chemicals and reagents were of analytical grade.

### 4.2. Osteoclast Differentiation

Bone marrow cells were prepared by removing from the femora and tibiae of 6–8 weeks old ICR mice as previously described [43]. The bone marrow suspension was added to plates. After 24 h of culture, the non-adherent cells were collected, layered on a Ficoll-Hypaque gradient, and the interface cells were collected, washed, and resuspended in α-MEM containing 10% FBS. For the osteoclastogenesis experiments, the bone marrow-derived macrophages (BMMs) were plated into a 96-well plate at a density of 2 × 10^4^ cells/well in α-MEM with 10% FBS along with M-CSF (30 ng/mL). BMMs were treated with fucoidan in RANKL (20 ng/mL) and M-CSF (10 ng/mL).

Osteoclast formation was measured by quantifying cells positively stained with TRAP. Briefly, the cells were fixed in 10% formaldehyde for 10 min and stained for TRAP with naphthol AS-MX phosphate and tartrate solution. TRAP-positive multinucleated cells (MNCs) that contained three or more nuclei were scored. For measuring TRAP activity, the cells were fixed, dried, and then incubated with 100-µL substrate solution (3.7 mM of *p*-nitrophenyl phosphate and 10 mM of sodium tartrate in 50 mM citrate buffer, pH 4.6) at 37 °C for 10 min. Following incubation, the reaction mixtures were transferred into new plates containing an equal volume of 0.1 N NaOH [43]. Absorbance was measured at 405 nm using an ELISA reader (BioRad, Hercules, CA, USA).

### 4.3. Proliferation Assays

Cell viability was determined using the CellTiter 96^®^ AQueous One Solution Cell Proliferation Assay kit (Promega, Madison, WI, USA) following the manufacturers’ instructions. Briefly, after 2 days treatment with fucoidan, 20 μL of CellTiter 96^®^ AQueous One Solution Reagent was added to each well, and the mixture was incubated for 2 h at 37 °C. Absorbance of each well was determined at 490 nm using a 96-well microplate reader (BioRad).

### 4.4. RT-PCR Analysis

Total RNA was prepared using TRI reagent according to the manufacturer’s instructions. *c*DNA was synthesized from 1 μg of total RNA using SuperScript II Reverse Transcriptase (Invitrogen). Previously published gene-specific primers for TRAP, Cathepsin K, GAPDH [44], *c*-Src [45], NFAT*c*1, DC-STAMP [46], and MMP-9 were used to amplify 1 μL of cDNA. The PCR products were captured with a LAS 4000 image documentation system (Fuji; San Leandro, CA, USA) after separation on 1% agarose gel electrophoresis.

Quantitative real-time PCR (qPCR) was performed on an ABI 7500 Real-Time PCR System using SYBR Green dye (Applied Biosystems, Foster City, CA, USA). The following primers were used: TRAP, 5'-TCCCCAATGCCCCATTC-3' and 5'-CGGTTCTGGCGATCTCTTTG-3'; CathepsinK, 5'-GGCTGTGGAGGCGGCTAT-3' and 5'-AGAGTCAATGCCTCCGTTCTG-3'; MMP-9, 5'-AAAGACCTGAAAACCTCCAACCT-3' and 5'-GCCCGGGTGTAACCATAGC-3'; NFATc1, 5'-ACCACCTTTCCGCAACCA-3' and 5'-TTCCGTTTCCCGTTGCA-3'; DC-STAMP, 5'-CTTCCGTGGGCCAGAAGTT-3' and 5'-AGGCCAGTGCTGACTAGGATGA-3'. Forty cycles of amplification comprised 10 min of denaturation at 95 °C that was followed by 15 s annealing at 95 °C, and finally 1 min extension and detection at 60 °C. All reactions were run in triplicate samples. The unknown samples were quantified by measuring the fractional cycle number (*C*_t_) against a standard curve. Briefly, all the target samples were normalized to a housekeeping gene, GAPDH. Then, the relative quantization value for each target gene compared to the calibrator for that target is expressed as 2-(*C*_t_ − *C*_c_) (*C*_t_ and *C*_c_ are the mean threshold cycle differences after normalizing to GAPDH). The relative levels of mRNA expression levels were plotted on a semi-log graph.

### 4.5. Resorption Pit Assay

For the resorption pit assay, mouse BMM cells (3 × 10^4^ cells/well) were seeded on dentine slices and treated with RANKL (20 ng/mL) and M-CSF (10 ng/mL) until multinucleated osteoclasts were formed. After the osteoclasts had formed, these cells were treated either with or without 0.5 μg/mL fucoidan in the presence of M-CSF and RANKL for 24 h. Then, the dentine slices containing the osteoclasts were stained for actin ring formation. Briefly, cells were fixed in 4% paraformaldehyde and permeabilized with 0.1% Triton X-100. After washing with PBS, F-actin was stained with TRITC-conjugated phalloidin (Sigma-Aldrich). The distribution of actin ring was visualized and detected under a LSM5 confocal microscope (Carl Zeiss, Jena, Germany). Following imaging, the cells were completely removed from dentine slices by abrasion using cotton tips and stained with hematoxylin solution to visualize resorption lacunae.

### 4.6. Western Blotting

Cell lysates were prepared using an extraction buffer composed of 50 mM Tris, pH 7.4, 1% NP-40, 150 mM NaCl, 1 mM EDTA, 1 mM PMSF, 1 mM Na_3_VO_4_, 1 mM NaF, 1 μg/mL pepstatin, and 1 μg/mL aprotinin. Protein concentrations were determined by Bicinchoninic Acid (BCA) protein assay (Pierce, Rockford, IL, USA). Approximately 30 μg of lysates was separated by 8%–12% SDS-PAGE, transferred onto a polyvinylidene difluoride (PVDF) membrane, and Ponceau S staining confirmed the equal transfer of the proteins. After transfer, the membrane was blocked in blocking buffer (5% non-fat dry milk in 20 mM Tris-Cl, pH 7.6) for 2 h. Proteins were detected by incubation with primary antibodies against c-JNK, phospho-JNK, ERK, phospho-ERK, p38 MAPK, phospho-p38 MAPK, phosphorylated IκBα, IκBα, NFATc1, c-Fos and β-actin, followed by incubation with secondary antibodies conjugated to horseradish peroxidase. Immuno-complexes were visualized by a chemiluminescence reaction using ECL reagents (Amersham Pharmacia Biotech, Buckinghamshire, UK).

### 4.7. Translocation of NF-κB

Mouse BMM cells were seeded at 1 × 10^5^ cells/well into a 8-well chamber slide. Adherent cells were transfected with 1.5 μg of the LigandLink™ pLL-1-NF-κB p65 GFP vector (Active motif, Carlsbad, CA, USA) using the Mirus reagent (Mirus Bio Co., Madison, WI, USA). Twenty-four hours post transfection, the cells were stained with LigandLink™ Fluorescein. BMMs were pre-treated with 5 μg/mL fucoidan for 30 min, and then stimulated with RANKL (20 ng/mL) in the presence of fucoidan for 30 min. The distribution of NF-κB GFP localization was visualized under an LSM5 confocal microscope (Carl Zeiss).

### 4.8. Statistical Analysis

A parametric one-way analysis of variance (ANOVA) was used to test for any difference among the groups. Tukey’s multiple comparison test was used to confirm significant differences among the group means. A *p*-value of less than 0.05 was considered significant.

## 5. Conclusions

In conclusion, the present study demonstrated that the fucoidan, a sulfated polysaccharide extracted from brown seaweeds, suppressed osteoclast differentiation and bone resorption activity at the early stage of osteoclastogenesis in cultured BMMs. Fucoidan displayed anti-osteoclastogenic potential by inhibiting MAP kinase and NF-κB, and down-regulating the expression of genes that were involved in both osteoclast differentiation and resorption.
